# Alleviation of acute radiation-induced bone marrow failure in mice with human fetal placental stromal cell therapy

**DOI:** 10.1186/s13287-020-01850-0

**Published:** 2020-08-03

**Authors:** Evgenia Volinsky, Astar Lazmi-Hailu, Nerel Cohen, Boaz Adani, Mohammad Faroja, Myriam Grunewald, Raphael Gorodetsky

**Affiliations:** 1 Laboratory of Biotechnology and Radiobiology, Hadassah - Hebrew University Medical Center, POB 12000, 91120 Jerusalem, Israel; 2 IMRIC-Developmental Biology and Cancer Research, Hebrew University School of Medicine, P.O. Box 12271, 91121 Jerusalem, Israel; 3 General Surgery, Hadassah – Hebrew University Medical Center, Jerusalem, Israel

**Keywords:** Acute radiation syndrome (ARS), Fetal human placental stromal cells (f-hPSCs), Hematopoiesis, Hematopoietic stem cells (HSC), Extra-medullary hematopoiesis (EMH), Bone marrow, Spleen, C3H mice

## Abstract

**Purpose:**

Selected placental mesenchymal stromal cells isolated from the fetal mesenchymal placental tissues (f-hPSCs) were tested as cell therapy of lethal acute radiation syndrome (ARS) with bone marrow regeneration and induced extramedullary hematopoiesis.

**Methods and materials:**

f-hPSCs were isolated from the chorionic plate of human placentae and further expanded in regular culture conditions. 2 × 10^6^ f-hPSCs were injected on days 1 and 4 to 8-Gy total body irradiated (TBI) C3H mice, both intramuscularly and subcutaneously. Pre-splenectomized TBI mice were used to test the involvement of extramedullary spleen hematopoiesis in the f-hPSC-induced hematopoiesis recovery in the TBI mice. Weight and survival of the mice were followed up within the morbid period of up to 23 days following irradiation. The role of hematopoietic progenitors in the recovery of treated mice was evaluated by flow cytometry, blood cell counts, and assay of possibly relevant growth factors.

**Results and conclusions:**

The survival rate of all groups of TBI f-hPSC-treated mice at the end of the follow-up was dramatically elevated from < 10% in untreated to ~ 80%, with a parallel regain of body weight, bone marrow (BM) recovery, and elevated circulating progenitors of blood cell lineages. Blood erythropoietin levels were elevated in all f-hPSC-treated mice. Extramedullary splenic hematopoiesis was recorded in the f-hPSC-treated mice, though splenectomized mice still had similar survival rate. Our findings suggest that the indirect f-hPSC life-saving therapy of ARS may also be applied for treating other conditions with a failure of the hematopoietic system and severe pancytopenia.

## Introduction

Acute radiation syndrome (ARS) following high-dose total body irradiation (TBI) is a severe life threatening condition [1–4]. The most severe complications are associated with the damage to the bone marrow (BM) and the hematopoietic system [2, 3]. Limited therapeutic approaches are currently available to treat the lethal hematopoietic ARS. Common treatments, such as matched hematopoietic stem cells (HSCs) transplantation [5], are not practical in circumstances associated with accidental high-dose exposure.

Mesenchymal stromal cells (MSCs) isolated from different tissues, primarily from the bone marrow, have raised interest in the field of regenerative medicine in the last decades [6–8]. Their therapeutic properties seemed to derive mostly from their paracrine activity [9–11]. Human placentae are an easily accessible source of fully differentiated placental stromal cells (PSC). Typically, the digestion of large placental tissues yields PSC mainly from the maternal placental tissue, occasionally with a few cells from the fetal placental tissues [12]. The fully differentiated adult PSC have a stable phenotype without spontaneous trans-differentiation. Some studies have shown their pro-regenerative and anti-inflammatory effect without serving as potential building blocks of damaged tissues [13, 14]. The ability of injected expanded human placental cells to mitigate ARS was shown earlier, where a mixture of maternal and fetal placental cells out-performed the effect of cells isolated from the maternal placental tissues only, with better effect on the induction of bone marrow regeneration [15]. Previous reports proposed the use of the immune privileged fetal stromal cells from Wharton Jelly of the placenta for the mitigation radiation damages of lower doses, which hints at the higher pro-regenerative and anti-inflammatory effect of the cells from the fetal placental tissues [16, 17]. Cell therapy based on the fetal stromal cell implants seemed to have beneficial effect, surpassing the negligible effect of the injections of G-CSF only [18].

Based on these insights, we developed a simple procedure for the isolation and expansion of the most effective fetal mesenchymal stromal cells, which we termed f-hPSCs, from the chorionic plate of the fetal placenta [12]. The biological rational of using the f-hPSCs is that the neonate chorionic plate tissues of the placental share the fetus circulation during pregnancy. Therefore, the cells in these fetal placental tissues are expected to respond to the fetal stress signals to which they are exposed by the secretion of factors that may assist controlling the fetal homeostasis [19–21].

The f-hPSC express mesenchymal stromal cell markers [15], as well as CD166, a common marker of mesodermal cell types and MSCs [22, 23] and CD146 (MCAM), which is expressed in different mesenchymal cells, including pericytes. The negligible expression of HLA-G (class I) and HLA-DR in f-hPSCs may contribute to the immune tolerance to these cells in both allogeneic and xenogeneic injections [24, 25].

We report here that remote injections of f-hPSCs, consisting predominantly of fetal placental cells, both by intramuscular (IM) or subcutaneous (SC) injections, following TBI with high dose of 8 Gy, most efficiently mitigated ARS with highly significant increase in mouse survival. The f-hPSC treatment enhanced BM hematopoiesis and promoted supplementary extra-medullar spleen hematopoiesis (Spleen-EMH), which seemed to contribute to the f-hPSC-induced recovery of the mice from lethal hematopoietic failure.

## Materials and methods

### Isolation, expansion, and characterization of the f-hPSCs

The f-hPSCs were isolated from placentae obtained from healthy mothers bearing healthy normal full-term male offspring after cesarian section as previously described [12]. The use of the donated placentae, with the full informed consent of the mothers, was approved by the Institutional Ethical Committee of the Hadassah-Hebrew University Medical Center (HMO-0361-14). This method of the f-hPSC isolation is based on the ability of f-hPSCs to migrate out spontaneously from the selected tissue fragments of ~ 1–2 mm which were cut from bulk tissue samples of the chorionic plate and adhered to the plastic culture plate with diluted fibrin, as previously described in detail [12]. By this isolation protocol, only pure population of f-hPSCs migrated out from the tissue fragments of the chorionic plate to the plastic dish to be further expanded. Within 2–3 weeks, a homogenous monolayer cell culture was obtained, which was further expanded by 6–8 cell passages before their collection for injection. Verification of the f-hPSCs origin as the placental stromal cells of the male newborn was done earlier, in cells from passages 3–4, by fluorescence in situ hybridization (FISH) of the centromeres of the X-Y chromosomes, showing ~ 100% of X-Y containing male cells. The pure mesenchymal nature of f-hPSCs was verified by examining the expression of typical stromal, hematopoietic, and endothelial cell protein markers by flow cytometry (MACSQuant, Miltenyi Biotech). The f-hPSCs did not express any hematopoietic or endothelial markers but did express typical stromal cells markers [12].

### Mice

Female C3H/HEN-HSD 8–9-week-old mice were used by approval of The Hebrew-University Medical School Institutional Animal Welfare Ethical Committee (MD-16-14727-4). The mice were kept throughout all the experiments in the animal facility under specific pathogen-free conditions (SPF) and were monitored and weighted 5–6 days a week. Mice’s health condition was assessed based on the “IBD Scoring table” provided by the ethics committee, allowing scoring the animal’s morbidity throughout the experiment. This includes the weight loss, mobility, and body posture of stress. Once the scoring of the mouse reached a low critical score, it was excluded and euthanized to avoid prolonged suffering.

### Irradiation

After the required acclimatization period of > 3 days at the animal colony, the mice were TBI at the radiotherapy unit of the Sharett Institute of Oncology at Hadassah Hospital in SPF conditions with photon beam of clinical 6 MeV LINAC (Varian, Medical Systems, CA, USA). The mice were placed in a plastic restricting Plexiglas jig, and 8 Gy was delivered through 5-mm plastic build-up for homogenous dose distribution. The dose was calibrated by the dosimetry physicists.

### Treatments with f-hPSC injections

The cultured f-hPSCs for the xenogeneic treatments to mice underwent 5–8 passages and were harvested for injection from confluent flasks using Trypsin-Versene EDTA solution (Biological Industries (BI), Israel). The cells were re-suspended in medium, counted, and centrifuged by 1400 csf for 5 min at 4 °C. Then the f-hPSCs were re-suspended in plasmaLyte A (designated also as “Vehicle”) to reach a final concentration of 2 × 10^6^ cells/100 μl. Two 50-μl injections of 10^6^ cells were delivered in each treatment intramuscularly (IM) or subcutaneously (SC), 1 and 4 days following 8-Gy TBI. In the IM treatment, the cells were injected to the large muscles of both hind legs, and in SC treatment under the skin of the upper back midline at the same time points, Veh-Cont mice were injected IM with the same volume of the vehicle solution.

### The experimental groups of mice tested

A total of 176 mice were included in this study. The treated TBI mice were injected with the cells twice, at 1 and 4 days following 8-Gy TBI. The IM injected (IM) (*n* = 39) or SC injected (SC) (*n* = 21). The non-treated control 8-Gy TBI mice (Veh-Cont) were injected at the same time points with the vehicle solution only. Due to the high mortality rate of the Veh-Cont group which were not treated with f-hPSCs, a higher number of mice were included in this group (*n* = 78) to assure the survival of a significant number of mice by the end of the follow-up for further monitoring different parameters tested. A fewer mice were used in the group designated as Naïve controls, which were not irradiated and were not injected with f-hPSC, which all survived with no observed adverse effects (*n* = 22). An additional group of TBI and f-hPS-treated mice were pre-splenectomized a week before irradiation ([Spl-]) (n= 13) to monitor the involvement of Spleen-EMH in the hematopoiesis recovery following f-hPSC treatment.

### Splenectomy

The surgical splenectomy of mice in the [Spl-] group was performed by a qualified surgeon at the animal’s facility laboratory in sterile conditions in the SPF facilities. The mice were fully anesthetized using a Ketamine-Xylazine cocktail. A 1–1.5-cm-long incision was made in the skin and subcutaneous left lateral subcostal tissue. The underlying muscle and peritoneum were pulled to expose the spleen in the left upper quadrant of the abdominal cavity. The spleen was carefully mobilized, and splenic vessels were ligated with resolvable fine sutures, while the gastro-splenic ligament was tied off. Then, the omentum was put in place, and the abdominal wall was closed with resolvable stitches. The mice were IP injected with Rymadil (4.4 mg/kg) in normal saline (0.7 ml) and were kept warmed until their full recovery from the anesthesia and then returned to their cage. About 10 days following splenectomy, the mice, which fully recovered from the operation with complete healing of the operation site, were TBI.

### Harvesting cells from the spleen and BM

At the termination of the follow-up, the spleen was extracted and weighed. For flow cytometry analysis, the cells of the mashed organs were sieved through a 40-μm strainer. Then, the extracted cells were counted and assayed for the relevant surface antigens.

BM cells were extracted from the four long bones (both tibiae and femurs), crushed with the use of mortar and pestle in buffer solution. The collected cells were filtered through a 40-μm mesh.

### Analyses of BM and spleen cells by flow cytometry

Single-cell suspensions obtained from BM and spleen were washed in staining buffer (0.2% BSA in PBS), and 2 × 10^6^ cells per sample were re-suspended in the staining buffer. Flow cytometry was performed on a MACS Quant Analyzer (Miltenyi), and analysis was completed on FCSexpress 4.0.

Flow cytometry analysis for assessing erythroid progenitor differentiation was done as follows: After exclusion of cells positive for other lineages (CD41^−^, B220^−^, CD3^−^, CD11b^−^, and Gr1^−^), the Ter119^hi^ population was gated to identify erythroid progenitors.

Forward scatter (FSC) and CD71 expression of the Ter119^hi^/Lin^−^ population subdivided erythroid progenitors according to their differentiation status into different subgroups as follows: EryA (basophilic erythroblasts), EryB (late basophilic erythroblasts), and EryC (orthochromatic erythroblasts/reticulocytes). HSCs were identified as lineage negative (TER119^−^, CD11b^−^, Gr1^−^, CD3^−^, B220^−^) and positive to cKit, Sca1, and CD150 [26]. Antibodies were purchased from Applied Biolegend (USA).

### CBC and reticulocytes counts

At the end of the experiments, the mice were deeply anesthetized and blood was collected directly by a fine needle from the heart into collection tubes with anti-coagulant. CBC was measured with COULTER® LH-750A Hematology Analyzer, Beckman Coulter, Inc. (Nyon, Switzerland), which was calibrated for murine blood cells. Proportions of circulating reticulocytes with residual cellular nucleic acids in the cell cytoplasm were detected in blood smears by modified Wright’s stain.

### ELISA of human and mouse proteins of interest

Plasma taken on day 7 following TBI was separated from the blood by centrifugation (2600 csf, 20 min) and stored at − 20 °C. ELISA essay was performed with preloaded kits for the different proteins, VEGF, EPO, and human/ murine PLGF (Quantikine Colorimetric Sandwich ELISA Kits, D&D Systems).

### H&E histology of bones and spleen

Tissue samples for histology were immersed for 24 h in formaldehyde 4% at room temperature and then transferred to 70% ethanol and processed for paraffin embedding. Fixed bone samples were fully decalcified for 4 days using a solution of EDTA (10%, pH 7.4) at 4 °C washed in running water and processed for embedding in paraffin. Then, 4-μm sections were processed for H&E and immunohistochemistry staining.

### Immune-histology of frozen sections of the liver for detection of hematopoietic sites

Livers were extracted from the euthanized mice and immersed in freshly prepared 4% paraformaldehyde, then transferred to 30% sucrose for overnight storage at 4 °C. Samples were then embedded in OCT compound, kept at − 80 °C, and cut with a thin section cryostat in 10-μm sections. The slides were stained for TER-119 (CY-3) and DAPI and kept at − 20 °C for fluorescence microscopy.

### Statistical analyses

Statistical analyses for the comparison between the different groups of interest were performed with Student’s two-sample *t* tests, assuming equal variances and by one-way ANOVA tests, where applicable. The significance of the difference between the survival curves was analyzed by a Log-Rank test of the Kaplan–Meier survival curves for both the survival duration and for the endpoint survival rate following different treatments. The *p* values are indicated within the graphs only where the difference between the groups tested was found to be significant. The error bars shown in the different figures represent standard errors of the mean (SEM).

## Results

### f-hPSC treatment in 8-Gy TBI mice dramatically improves their survival and weight recovery

**Fig. 1:**
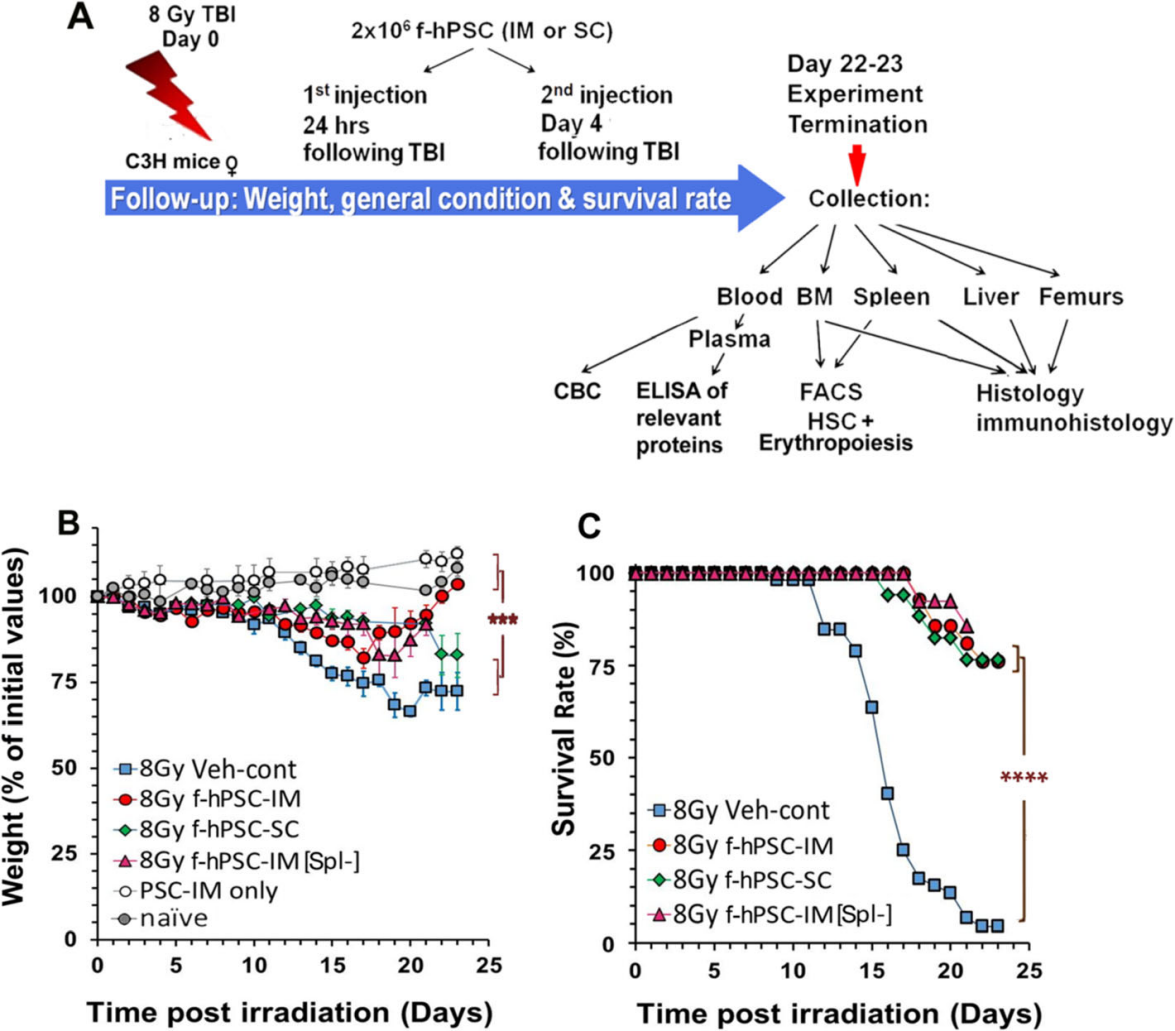
Experimental set-up and follow up of mice weight and survival. **a** Experimental set up. TBI of 8 Gy was done on day 0. The 2 × 10^6^ f-hPSCs were injected IM (IM-f-hPSCs) or SC (SC-f-hPSCs) on days 1 and 4. Pre-splenectomized mice [Spl-] were treated only with IM f-hPSC injections. Weight and survival were followed up for up to 23 days (**b**, **c**, respectively). Non-irradiated f-hPSC-treated and non-treated Naïve mice served as controls

The experimental plan of the current study is shown in Fig. 1a. TBI-induced mortality is observed in our model only within the first ~ 20 days following the 8-Gy TBI. At the first 9 days, a similar degree of moderate weight loss was observed in all the TBI groups (Fig. 1b). From then on, the weight loss in Veh-Cont group persisted with a death toll of about > 90% of the mice within 7–20 days from irradiation (Fig. 1c). In all the f-hPSC-treated TBI groups, nearly 80% of the mice survived and almost fully regained their lost weight by the end of the follow-up. But the regain of body weight was slower in the [Spl-] group. Though there was no significant difference in the survival rate between the different f-hPSC-treated groups, the IM treatment was found to be most effective in terms of general recovery of the mice, as reflected by the follow-up of weight loss and gain (Fig. 1b). This is best demonstrated at the end of the experiment, where the SC-treated mice had significantly lower weight regain than IM treated, though the overall survival rate was similar.

### Blood cell counts recovery following f-hPSC treatment

**Fig. 2:**
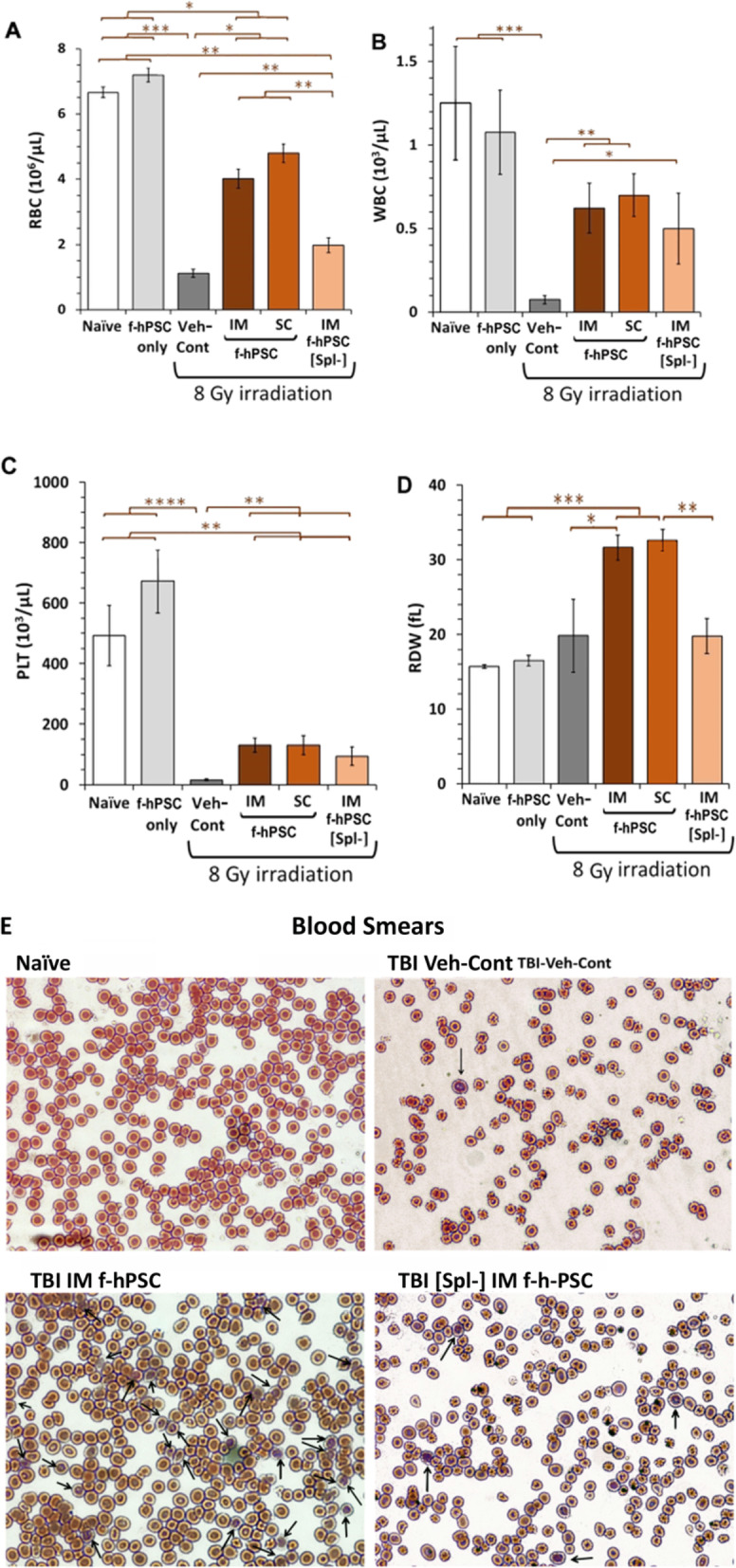
The CBC profile of the survivors at the termination of the follow-up. WBC, RBC, and PLT counts and RDW were measured at the end of the follow-up for all the experimental groups tested. **a**–**d** Giemsa stained blood smears detect prematurely released reticulocytes to the circulation (**e**) (*p* value: *< 0.05, **< 0.01, ***< 0.001, ****< 10^−3^)

The complete blood cell counts (CBC) for the different groups tested were measured at the end of the follow-up, before a further hematopoiesis reconstitution could mask these differences. Leukocytes (WBC) and erythrocytes (RBC) counts were significantly elevated in TBI f-hPSC-treated mice and approached the values of non-irradiated Naïve mice (Fig. 2a, b). The platelet counts in f-hPSC-treated TBI mice were significantly recovered relative to Veh-Cont, but were still lower than those of the Naïve group (Fig. 2c). In spite of the similar survival rate, the [Spl-] group had lower counts of RBC, WBC, and PLT than those of the f-hPSC-treated TBI groups (Fig. 2a–c), hinting for an additional contribution of Spleen-EMH to the hematopoietic recovery in the TBI and f-hPSC-treated group.

The RBC size distribution width (RDW) index was higher in f-hPSC-treated TBI mice than in Naïve or TBI Veh-Cont. Interestingly, this parameter was not elevated in the [Spl-] group (Fig. 2d). This suggests that f-hPSCs induced accelerated RBC repopulation, releasing premature larger RBC progenitors, possibly with contribution of the Spleen-EMH. Giemsa stained blood smears supported these findings, and showed a few immature nucleated blood reticulocytes in the circulation of the Naïve and Veh-Cont groups and highest number in IM f-hPSC-treated TBI mice. The fact that low numbers of such cells were seen in the [Spl-] group further hints that a great part of the reticulocytes in f-hPSC-treated TBI mice derived from the Spleen-EMH (Fig. 2e).

### f-hPSCs induced contribution of BM and Spleen-EMH to hematopoietic recovery

**Fig. 3:**
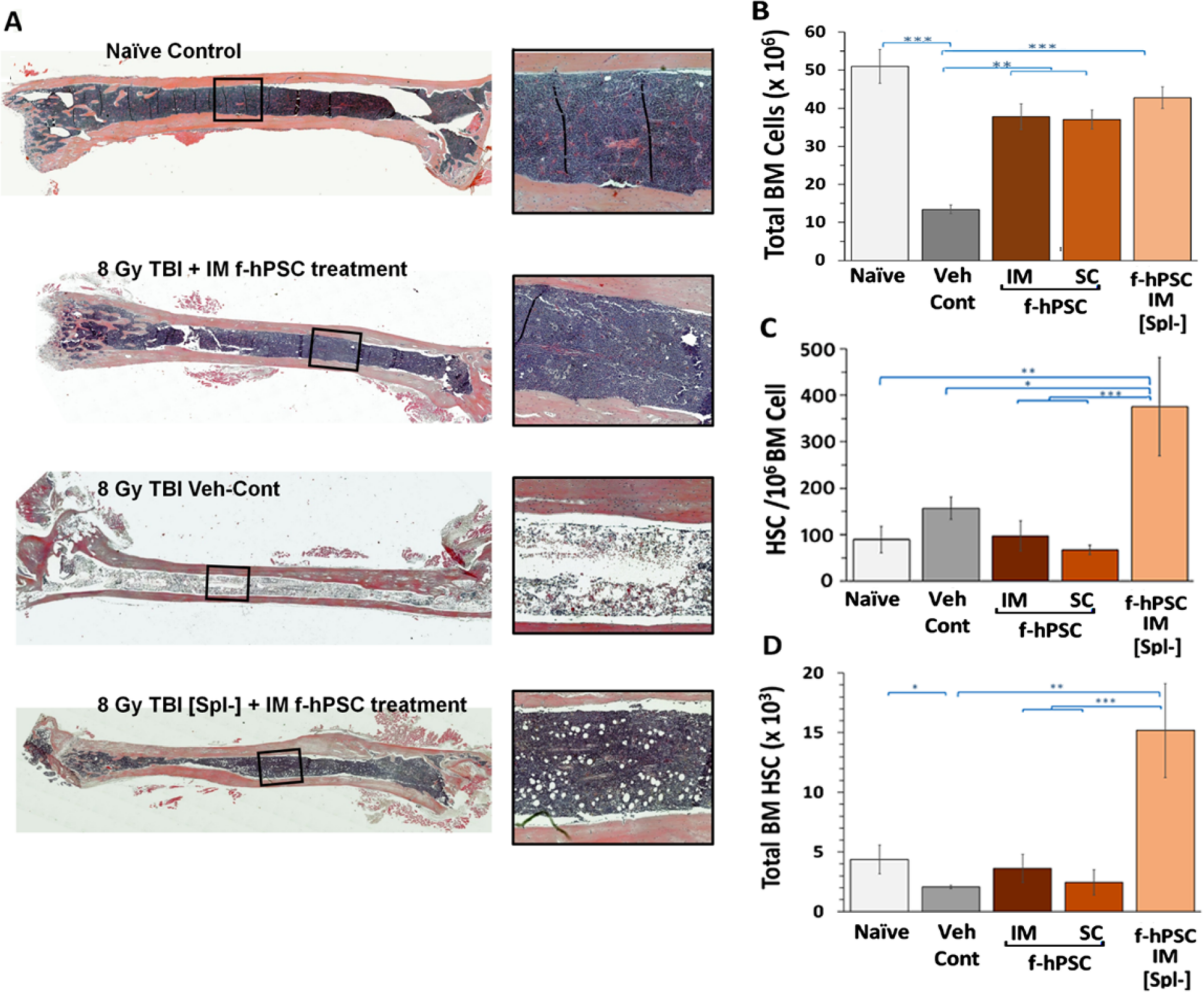
Histological sections of the bones and BM cellularity of the different groups tested. Representative cross-sections of tibia of mice at the termination of the follow-up stained with H&E to observe the degree of regeneration of the BM (**a**). A typical area in each bone section is further magnified. The difference in the cellularity of the BM in each group is demonstrated. Total BM cell counts obtained from the 4 long bones of all mice, frequency of HSCs, and total numbers of HSCs in the different groups are presented in (**b**–**d**), respectively

f-hPSC induction of BM regeneration corresponded well with the survival rate of the different experimental groups tested. Cell density in the BM tissues is demonstrated in histological cross-sections of the tibiae at the termination of the follow-up. The cell density in all f-hPSC-treated groups, including the [Spl-], showed nearly a full recovery of the cellular BM compartment at the end of the follow-up, approaching the BM density of non-irradiated Naïve mice. In contrast, in the few most resilient mice of the Veh-Cont group which survived to the end of the follow-up, the significantly depleted and fibrotic BM was seen in the bone section, with increased adipose tissue infiltration (Fig. 3a).

The total BM cell number from the long bones confirmed the reduced cellularity in the untreated Veh-Cont group relative to Naïve and the f-hPSC-treated groups (Fig. 3b). The frequency of HSCs in the surviving Veh-Cont mice relative to the total number of BM cell derives from the most extreme stress on the BM hematopoiesis in these few survivors, which persisted to end of the follow-up. Since their extracted total BM cell number was very low, the proportion of the HSCs which may account for the eventual survival of these few mice, relative to the low total BM cellularity in these few surviving mice, was somehow elevated. The [Spl-] group reached the highest values, possibly due to the lack of supporting Spleen-EMH compartment in the splenectomized mice (Fig. 3c). Still these findings are not reflected in the follow-up of survival rate of the [Spl-] group, which is not significantly different in [Spl-] relative to other irradiated and f-hPSC-treated groups (Fig. 1c).

**Fig. 4:**
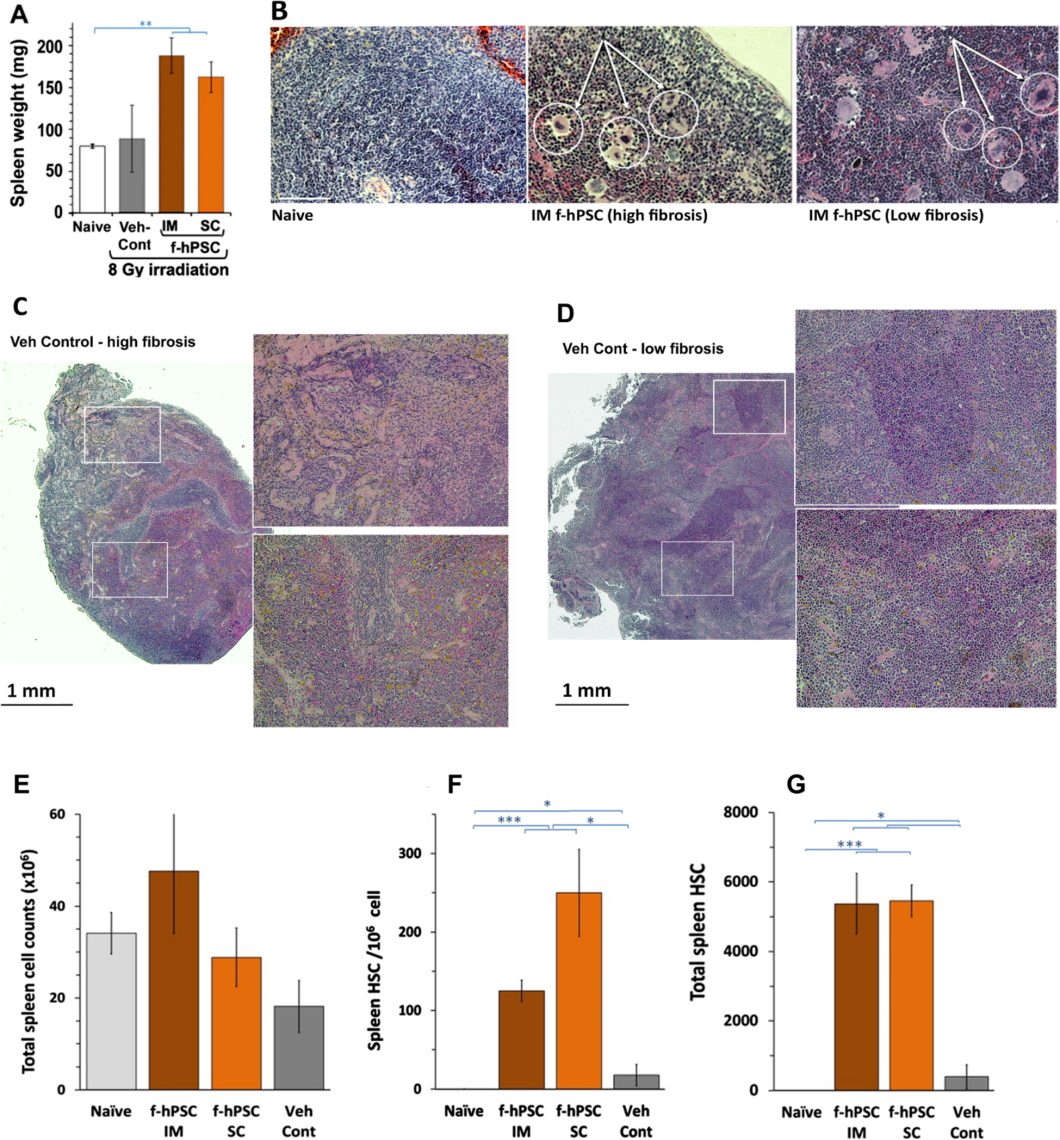
Spleen and liver sites of extramedullary hematopoiesis. **a** Spleen weight at the termination of the follow-up. Increased spleen weight was associated with elevated number of megakaryocytes only in TBI IM or SC f-hPSC-treated mice, as compared to the naïve controls (**b**). The spleen cellularity and size, which may represent the extent of spleen damage which varied in the few surviving Veh-Cont mice, are presented (**c** Vs **d**, with magnification of selected area). Increased total number of spleen cells and the presence of HSCs (identified as Lin- negative, [cKit+, SCA1+ and CD150+) in f-hPSC-treated mice, as compared with Veh-Cont and Naïve mice, may suggest increased Spleen-EMH. Total numbers of isolated cells, HSC frequency, and their total counts in the spleen are presented in **e**–**g** (*p* values:*< 0.05, **< 0.01, ***< 0.001, ****< 0.0001)

The observation that the survival rate of f-hPSC-treated [Spl-] mice was not significantly different from the other TBI f-hPSC-treated groups (Fig. 2b) suggests that splenic hematopoiesis may have contributed to the recovery of the mice but is not critical to the hematopoietic recovery of the TBI f-hPSC-treated mice. The higher number of HSCs in the BM of the f-hPSC-treated group suggests that the loss of the potential additional contribution of the spleen is compensated by the increased rate of hematopoiesis in the BM (Fig. 3c, d). The spleen of f-hPSC-treated TBI mice were found to be significantly enlarged relative to both Naïve and TBI Veh-Cont groups (Fig. 4a). Megakaryocytes, which may indicate the existence of active Spleen-EMH and indication of extramedullary hematopoiesis, were identified in histological splenic sections of TBI f-hPSC-treated mice (Fig. 4b), but were not seen in the Naïve group. The spleen in the few surviving Veh-Cont mice was either highly fibrotic (Fig. 4c) and/or slightly enlarged (Fig. 4d), with no apparent megakaryocytes. These findings were partially supported by an increase in total number of cells isolated from the spleen in the f-hPSC-treated groups, as compared to Naïve mice and to Veh-Cont (Fig. 4e). The total number of HSCs in the spleen was not significantly different in the different groups, though it was somehow reduced in the Veh-Cont (Fig. 4e). No HSCs were identified in the spleen of the intact Naïve mice, and only very few such cells were seen in the untreated TBI Veh-Cont group (Fig. 4f, g). The f-hPSC treatment significantly elevated the number and proportion of HSCs in the Spl, with the contribution of to the hematopoietic recovery. But the effect of the lack of this additional extramedullar hematopoietic compartment was not reflected in the survival rate of the [Spl-] group, which was not significantly different from the non-operated f-hPSC-treated TBI mice.

**Fig. 5:**
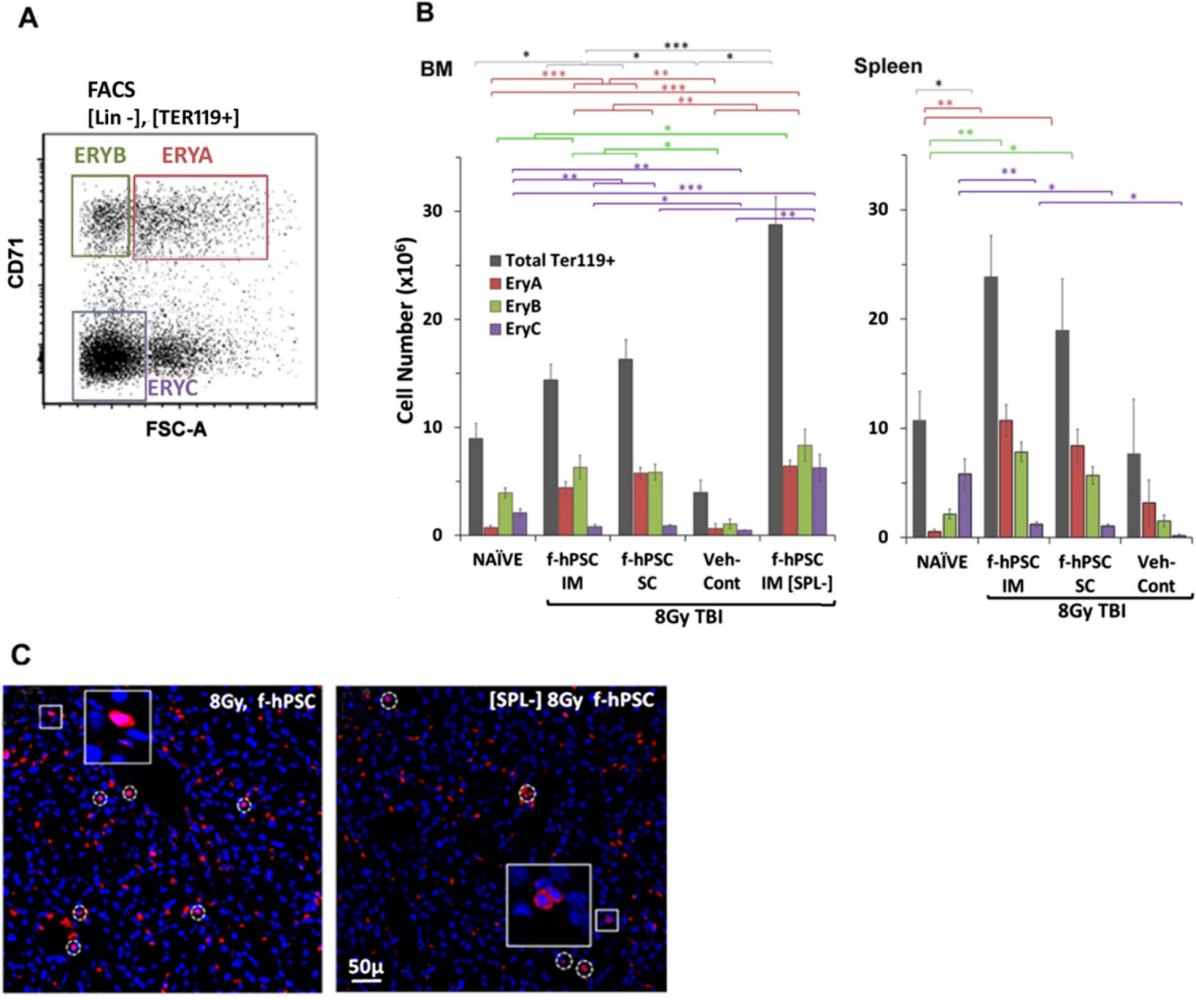
Maturation state of erythropoietic cells in the spleen and in the BM of the groups tested. **a** Flow cytometry of the different stages of erythropoiesis in the BM and spleen in mouse groups as tested with cell-markers CD71 and Ter119 [27]. *EryA* cells are less mature, large proliferating erythropoietic progenitors, both CD71^+^ and TER119^+^. *EryB* cells are smaller CD71^+^ cells. *EryC* representative is most mature small non dividing erythrocytes. Distribution of these cell populations harvested from the BM and spleen at the termination of the follow-up is presented in **b**. A comparison of Ter119 Cy3 nucleated cells staining of liver cryosections of non-operated and |Spl-] f-hPSC-treated TBI mice (**c**). Only a few individual cells (marked by a circle) where stained in the liver sinusoids, with no apparent liver extramedullary erythropoiesis, in spite of the higher hematopoietic stress in [Spl-] group due to the absence of spleen-EMH (*p* values: *> 0.05, **> 0.01, ***> 0.005)

Erythropoiesis was evaluated by the distribution of RBC progenitors by flow cytometry at the end of the follow-up. Cells from both the BM and the spleen were examined. This population gating according to cell size (as reflected in the Forward Cell Scatter parameter) and expression of Ter119 and CD71 (Fig. 5a) was used [27]. Both f-hPSC treatments boosted the regeneration of erythropoietic populations in the BM (Total Ter199+cells), as illustrated by a significant increase in the number of immature erythropoietic progenitors (EryA and EryB) in comparison to the Veh-Cont group. BM erythropoiesis was even further increased in the splenectomized mice, confirming the compensatory mechanism by the BM for the absence of the f-hPSC-induced Spleen-EMH contribution (Fig. 5b). The effective recruitment of the spleen in f-hPSC-treated arms to support erythropoiesis is demonstrated by the increased number of progenitors at different early stages of erythropoietic maturation (Fig. 5c).

Of interest is the lack of erythropoietic colonies in the liver of TBI mice, even in [Spl-] f-hPSC-treated mice, where Spleen-EMH could not contribute to hematopoietic recovery (Fig. 5c). These results indicate that even following f-hPSC treatment, ARS cannot induce recruitment of the liver tissues as an additional site of extramedullary erythropoiesis.

**Fig. 6:**
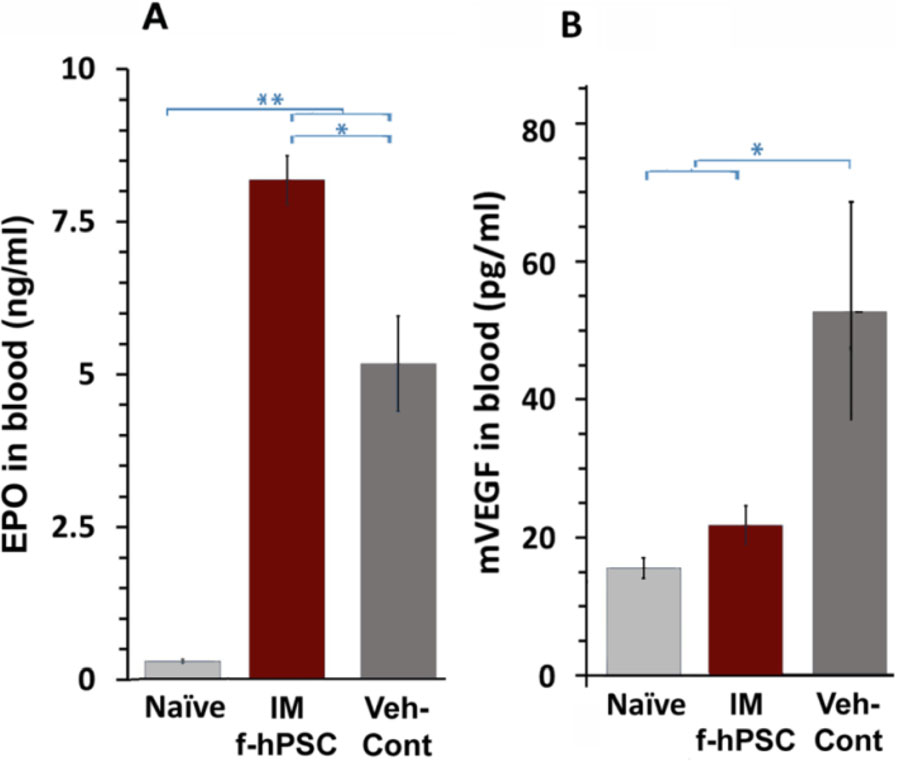
Plasma EPO and m-VEGF levels on day 23, at the termination of the follow-up. On day 7 of the follow-up, concentrations of human and murine EPO (**a**) and VEGF (**b**) were measured in the blood plasma of the TBI mice, treated or untreated with f-hPSCs. EPO level was significantly elevated in TBI mice and further increased in f-hPSC-treated mice. h-VEGF was not detected, but m-VEGF reached detectable levels and was significantly higher in the untreated TBI mice, relative to the Naïve or f-hPSC-treated TBI mice (*p* value: *< 0.05, **< 0.01)

Besides the expected systemically secreted mouse EPO in hematopoietic crisis, one could expect on day 7 in the most stressful phase of the mice a contribution of additional EPO secretion by the injected f-hPSCs of human origin. This expectation is based on previous reports that EPO could be secreted by the placentae [28]. We found that the total EPO was significantly elevated in the TBI mice at that time point, as compared to naïve mice. Highest EPO concentrations were detected in the f-hPSC-treated mice (Fig. 6a). This correlated well with the erythropoietic recovery observed in the f-hPSC-treated TBI mice. But due to high inter-species homology, the human f-hPSCs and murine EPO could not be detected separately by the ELISA assay, so the exact source of the circulating EPO could not be determined.

Based on previous findings of VEGF-induced erythropoiesis [29], both human and murine circulating VEGF levels (h-VEGF and m-VEGF, respectively) were assayed by ELISA on day 8, at the peak of the TBI-induced stress. No detectable levels of f-hPSC-derived h-VEGF were found in the blood of all the groups tested at that early time point. The level of m-VEGF was elevated significantly only in the circulation of the TBI Veh-Cont mice, suggesting that the m-VEGF induction by hypoxic stress was lower in the treated mice at this time point (Fig. 6b). In view of the effect of the TBI and treatment on VEGF levels, it is of interest that human and mouse placental growth factor concentrations (PLGF) [30] were both found below the detection levels in all the experimental arms tested (not shown).

## Discussion

Earlier proposed platforms of stem cell therapies with progenitors cells, either alone or loaded on scaffolds, were initially expected to function as building blocks of the damaged tissues [31, 32]. Typically, in most of the stem cell studies where a therapeutic effect for different medical conditions was reported by such cell therapy, a very limited evidence of a possible integration of the delivered cells to replace those of the affected damaged tissue was given. Typically, where such studies reported a significant effect, it seemed to be due to the cells’ secretome as indirect paracrine effect [24, 33–35]. The problem with some of such cell therapies is that they may also be potentially hazardous due to the undesired potential transformation of cells such as multi-potent MSC [36].

To overcome the limited supply of BM-MSC for cell therapies, adult cells from other mesenchymal tissues were tested for their indirect pro-regenerative and anti-inflammatory effects. This paved the way for allogeneic therapies with mesenchymal stromal cell types from various sources, where immune-tolerated and highly responding cells have an advantage [24, 37, 38]. The pro-regenerative activity of the f-hPSCs, which are fully differentiated adult cells and not stem cells by definition, seem to be based on their indirect trophic effects. In such circumstances, the issue of “stemness” is irrelevant to their indirect pro-regenerative effect [39, 40].

The IM and SC injections of the f-hPSCs seem to be an adequate alternative to the more hazardous IV injection, which may result in the immediate trapping of the cells in the lung with possible subsequent complications with no expectation for homing to specific damaged tissues and organs [15].

Since the fetal placental tissues co-reside during pregnancy with the non-tissue-matched maternal tissues, with minimal rejection, the f-hPSCs from this immune-tolerated tissue are expected to be less rejected in allogeneic or xenogeneic delivery with their extended residence in the injected sites. The exact location of the remote f-hPSC administration, either by IM or SC injections, does not seem to be crucial for their effect, as long as the cells are exposed to the stress messages carried in the blood circulation to the tissues to which they are injected. Still a follow-up of parameters of general health recovery, such as weight regain, the IM injected cells were somehow more effective than those delivered SC.

Our study demonstrates the pro-regenerative and highly significant life-saving effect of the remote injections of f-hPSCs following lethal irradiation. The highly significant mitigation of ARS was associated with the expedited induced BM regeneration and recovery of the hematopoietic system in the f-hPSC-treated mice. We could show that this process was aided by f-hPSC-induced Spleen-EMH, but was not critically dependent on it.

Since mortality was restricted to the first ~ 10-20  days following irradiation, by ~day 20 the fate of the irradiated mice was already determined, as previously demonstrated in studies with a similar mouse model [15, 41]. Our follow-up was terminated early,  on day 23, to allow the comparison of the hematopoiesis recovery between the groups tested, before this difference fully faded due to the progressive BM recovery of the surviving mice beyond this period (Fig. 1).

The critical BM precursor compartment injured by irradiation is the myelo-erythroid lineage. Except for the surviving TBI Veh-Cont mice, where the blood cell counts in the few survivors, especially the PLT, were still severely depleted at the termination of the follow-up (Fig. 2), a significant recovery of the f-hPSCs-treated mice seemed to be associated with the treatment-induced regeneration of their hematopoietic cell population. This resulted in a fast recovery of the RBC, WBC, and platelets counts in the f-hPSC-treated mice. This stress on the hematopoietic system in all f-hPSCs-treated TBI mice was associated with the release of immature larger reticulocytes and erythroblasts to the circulation, which was reflected in RBC size variability, as shown in elevated RDW index (Fig. 2d). This is a common observation following severe anemic conditions, where the BM hematopoiesis compensates for the low peripheral blood cell counts by a fast production and early release of immature reticulocytes to the circulation [42]. Of interest was the finding that the general health condition of mice in IM injected cells improved faster than the mice injected SC according the follow-up of weight regain. This may be due to the better exposure of the f-hPSCs to the circulation within the highly vascularized muscle relative to the subcutaneous tissues, allowing their better exposure to stress signals and subsequent release of pro-regenerative factors to the circulation to trigger the accelerated hematopoiesis regeneration.

The ability of the spleen to contribute to the hematopoiesis process by colony forming units of HSCs was already established in the early 1960s with the studies of Till et al. [43]. Therefore, we expected that the spleen may play a crucial role in the accelerated recovery in the f-hPSC-treated mice by providing an additional significant hematopoietic site in compensation to the depleted BM. Indeed, we found that the f-hPSC treatment triggered Spleen-EMH with notable splenomegaly in TBI mice. But this was not observed in Veh-Cont mice with more severe BM failure, suggesting that the f-hPSC treatment also induced EMH in the spleen.

The additional arm of TBI f-hPSC-treated [Spl-] mice further pointed to possible role of the spleen in the f-hPSC-induced mice recovery from ARS. Though the effect of f-hPSC treatment on the survival of this group was not significantly different from non-operated mice and was based on the effect of f-hPSC treatment on the regeneration of the bone marrow, the lower peripheral blood cell counts in the f-hPSC-treated arm of TBI [Spl-] hinted for the possible contribution of the Spleen-EMH to the better condition of the f-hPSC-treated TBI mice, as reflected by higher weight gain. The conclusion seems to be that f-hPSC treatment, either by IM or SC administration, mitigates hematopoietic ARS mainly by boosting the rate of BM regeneration with a limited reinforcement by the f-hPSC-induced spleen-EMH. These findings are clearly supported by the histology of bone sections at the end of the experiments, where the BM tissues in the long bones recovered almost completely in all the arms of the f-hPSC-treated mice, with much lesser BM regeneration in the few stronger survivors in the arm of Veh-Cont mice.

The liver seems to support hematopoiesis only in early fetal development [44]. But in spite of the stressful pancytopenia following TBI, we could not detect EMH in the liver in any of the experimental groups tested. 

The accelerated recovery of the hematopoietic system in f-hPSC-treated mice initiated 7–9 days following TBI and seemed to coincide with the elevation of EPO level in TBI Veh-Cont and the further elevation in the f-hPSC-treated mice (Fig. 6). Unfortunately, our ELISA immunoassay could not distinguish between the highly conserved human and the murine EPO homologues. Therefore, the source of the EPO could be either a part of the human factors secreted by the f-hPSCs or a treatment-induced murine EPO secretion, or both. Either way, it is anticipated that EPO secreted by f-hPSCs or indirectly by the irradiated mice accelerated hematopoiesis regeneration in the BM as well as in Spleen-EMH sites.

Radiation-induced stress is expected to increase also VEGF secretion [45, 46]. Elevated VEGF levels were recorded on day 7 only in the plasma of the untreated Veh-Cont mice and not in the recovered f-hPSC-treated mice. This may suggest a higher systemic stress in the Veh-Cont group at that critical phase. Of interest is that the placenta VEGF analogue PLGF [47], which is specific to placenta tissues, was not elevated in any of the groups tested (not shown).

Though our investigation demonstrates very clearly the highly significant effect of the f-hPSC treatment to promote bone marrow regeneration and prevent ARS, our further studies will try to explore in detail the mechanism behind the f-hPSC effect by gene expression profiles and proteomics of the implanted cells of the TBI mice.

In conclusion, we demonstrate the pro-regenerative mechanism of action of allogeneic or xenogeneic f-hPSC therapy for mitigation of lethal high dose TBI. The regimen of 2 f-hPSC injections, delivered either IM or SC, dramatically increased the survival of the TBI mice and supported a recovery towards their initial weight, with a significant recovery of peripheral CBC parameters. This seems to be the result of the treatment induced boosting the BM hematopoiesis, with possible limited contribution of spleen-EMH.

Therefore, we suggest that the highly potent expanded f-hPSCs derived from the fetal placenta (in our case: all male, of X/Y karyotype) may serve as an optimal non-hazardous allogeneic cell therapy, optimally suited to mitigate ARS in all species, including humans. The f-hPSC treatment as a simple and apparently non-hazardous cell therapy, with no apparent adverse effects or complications, should not be limited to treat only ARS. Rather, it could be considered also to individuals possibly exposed to sub-lethal doses, to enhance their recovery rate. We also suggest that this systemic treatment could be applied for the treatment of other conditions associated with severe BM failure and subsequent hematological crisis and pancytopenia.

## Data Availability

The data that support the findings of this study and calculations are available upon request from Prof. Raphael Gorodetsky, the corresponding author. Parties interested to review the data on which this publication is based could contact Prof. Raphael Gorodetsky at Hadassah Medical Center.

## Abbreviations

ARS: Acute radiation syndrome
PSC: Placental stromal cells
f-hPSCs: Fetal human placental stromal cells
TBI: Total body irradiated
Gy: Gray, radiation dose unit
HSCs: Hematopoietic stem cells
MSCs: Mesenchymal stromal (stem) cells
SC: Subcutaneous
IM: Intramuscular
EMH: Extra-medullary hematopoiesis
BM: Bone marrow
[Spl-]: Splenectomized TBI mice
CBC: Complete blood cell counts
Plt: Platelets
RBC: Red blood cells
WBC: White blood cells
SPF: Specific pathogen-free conditions
